# Exploring the mechanism of “Rare Earth” texture evolution in a lean Mg–Zn–Ca alloy

**DOI:** 10.1038/s41598-019-43415-z

**Published:** 2019-05-09

**Authors:** Dikai Guan, Xingguang Liu, Junheng Gao, Le Ma, Bradley P. Wynne, W. Mark Rainforth

**Affiliations:** Department of Materials Science and Engineering, University of Sheffield, Sheffield, S1 3JD UK

**Keywords:** Metals and alloys, Characterization and analytical techniques

## Abstract

The entire recrystallisation sequence and associated crystallographic texture evolution of Mg-0.8Zn-0.2Ca (wt.%) alloy was tracked using a *quasi-in-situ* electron backscatter diffraction (EBSD) method. Characteristic “Rare Earth” (RE) texture was formed, originating mainly from double twins and twinning-related shear bands consisting of compression and double twins. The RE textures appeared during the nucleation stage and were preserved during the relative uniform grain growth period because of solute segregation and concurrent precipitation although the alloying element content was very low. Ca and Zn co-segregated along grain boundaries with no evidence that segregation was preferred along special types of grain boundaries. The interactions between deformation microstructures, concurrent precipitation, solute drag, grain growth and texture evolution are discussed in detail. All the results indicate that Ca performs a similar role to that of RE elements in forming RE texture.

## Introduction

Although Mg alloys have low density and high specific strength, their application has been limited by poor formability at room temperature owing to the hexagonal close packed crystal structure. A strong basal crystallographic texture is also often developed after thermomechanical processing^1–5^ giving poor ductility and a strong yield asymmetry^6,7^. This texture cannot be effectively modified or eliminated in most conventional Mg alloys^8,9^. Recent studies have reported that the addition of rare earth (RE) elements can randomise the basal texture by forming characteristic RE textures, either during thermomechanical processing or on subsequent annealing^1,6,10–14^. Various mechanisms and hypotheses have been proposed to explain the formation of RE textures, such as the location of nucleation sites^2,4,6,11,12,15–18^, increased non-basal slip activity^5^, orientated grain growth generated by solute drag or particle pinning along specific types of grain boundaries (GBs)^19–23^. Among these disputed mechanisms, orientated grain growth controlled by solute drag has gained significant attraction^1,10,20,22^, as RE elements have a large atomic radius and segregate strongly in Mg-RE alloys. However, it is likely that several mechanisms are operating at once.

RE elements are expensive and strategically important and so there has been much effort to find alternatives. The alternative needs to impart similar effects to the RE containing alloys but allow a reduction in the production cost and thereby enhance the future viability of Mg alloys. Because of a large atomic radius Ca has attracted substantial attention as a candidate to replace RE elements. Indeed, weakened basal and RE textures have recently been obtained in Ca containing conventional Mg alloys without RE additions^19,24–34^. The texture modification and RE texture formation were attributed to several different mechanisms: particle stimulating nucleation (PSN)^26,34^, dynamic strain aging^28^, preferred growth of oriented nuclei^32^, solute drag^19,31^, modification of c/a ratio and stacking fault energy^27,33^. Zeng *et al*.^19^ systematically compared the texture evolution in Mg-0.1Ca, Mg-0.4Zn and Mg-0.3Zn-0.1Ca (at.%) during static annealing. A weakened texture was only found in the ternary alloy. They hypothesised that co-segregation of Zn and Ca atoms to the grain boundaries in the ternary alloy strongly restricted high-energy GBs mobility of the basal recrystallised grains. They also stated that the formation of the weakened texture was attributed to uniform growth of recrystallised grains originating from grain boundary recrystallisation, rather than shear band recrystallisation or deformation twin recrystallisation. This was because there was limited occurrence of both shear bands and deformation twins in the ternary alloy. On the other hand, weakened basal and RE textures have been shown to form in WE43 when the deformed microstructure had a high density of shear bands and deformation twins^18,35^, where grain boundary recrystallisation was largely restricted. Stanford^31^ has already shown that Ca can act in a similar manner to RE elements in texture strength evolution in extruded material because of its large atomic radius.

Despite these above mentioned studies, there remains a number of questions that need to be addressed. Ca owns a similar atomic radius with most RE elements and can weaken the basal texture, but is the role of Ca similar or different to RE elements to control the recrystallised texture during annealing? Can an RE texture form in Mg-Zn-Ca systems with a similar deformed microstructure as developed in the RE containing WE43 alloy^18^? Which stage from nucleation or grain growth is critical for the final texture formation? Solute segregation along grain boundaries has been commonly reported. High quality images collected by the widely used high-angle annular dark-field scanning transmission electron microscopy (HAADF-STEM) and coupled EDX can only tell us which alloying elements segregate along grain boundaries, but the corresponding grain boundary misorientation parameters cannot be extracted from these images. Therefore, the question remains as to whether RE elements or Ca segregate only to some special GBs or do they segregate universally?

To address these issues, the recrystallisation texture evolution of Mg-0.8Zn-0.2Ca (ZX10) was systematically tracked by a *quasi-in-situ* EBSD method through the entire recrystallisation sequence. Typical RE textures was produced in this alloy. Direct observation showed Ca and Zn co-segregated globally along various high angle grain boundaries. A concurrent precipitation occurred during recrystallisation process. These results implied Ca played an analogous role to that of RE elements in forming recrystallisation textures during annealing.

## Results

### Microstructure after cold rolling

**Figure 1:**
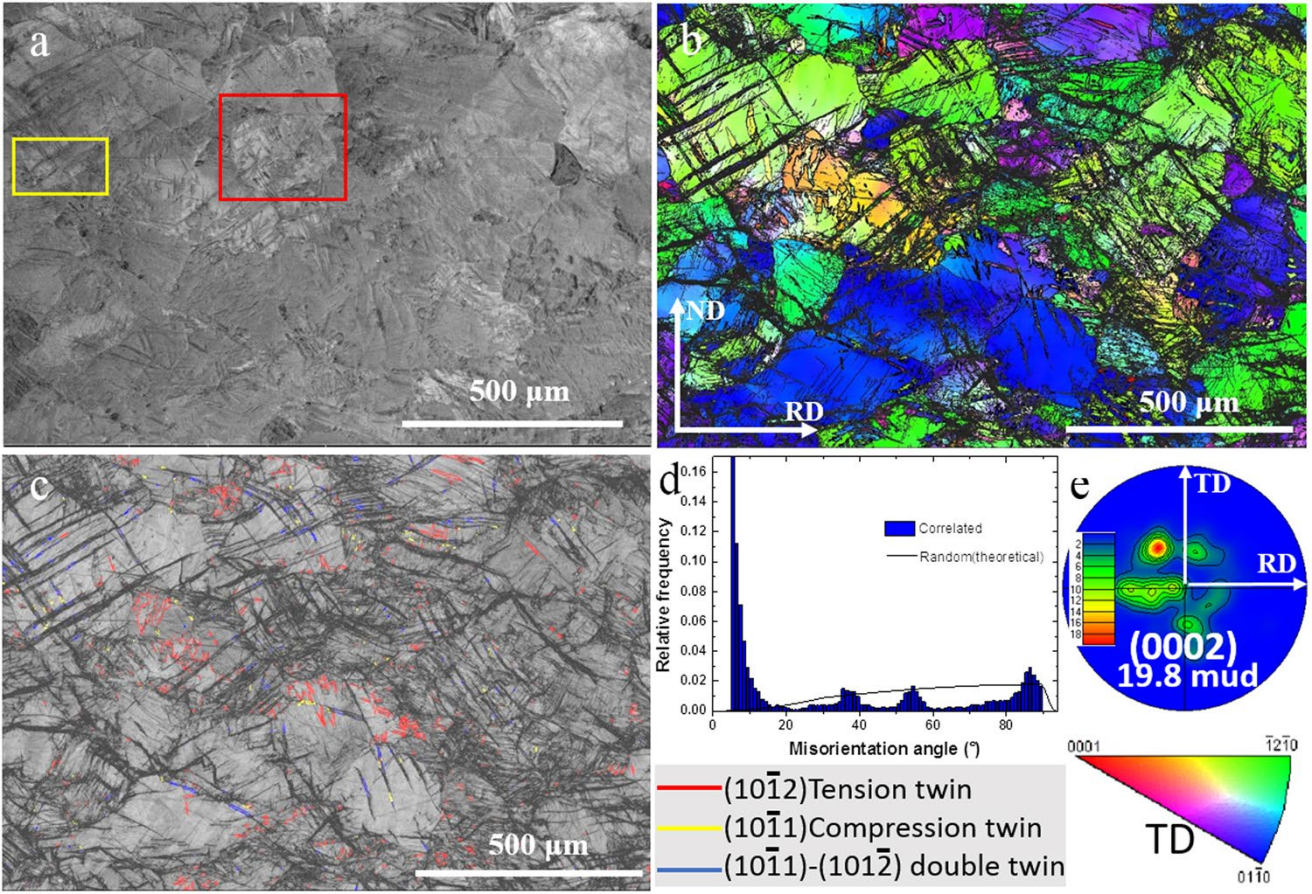
(**a**) Backscattered SEM image of cold-rolled ZX10, (**b**) corresponding EBSD IPF image, (**c**) band contrast (BC) map superimposed by various twin boundaries, (**d**) misorientation angle and (**e**) (0002) pole figure of (**b**). Observation along TD was applied to IPF triangle.

Figure 1(a) shows backscattered SEM (BSEM) images of a cold-rolled sample for *quasi-in-situ* EBSD investigation. No noticeable second phase particles were observed in this large sampling area (~1.4 mm^2^) nor in the high magnification BSEM images which are not presented here. The dimples on the sample surfaces were introduced by final argon ion polishing using a Gatan precision etching and coating system (PECS)^11^. Figure 1(b) shows inverse pole figure (IPF) map and the corresponding (0002) pole figures are presented in Fig. 1(e). An annular texture around the ND (out of the page) was observed 32.5 degrees from the ND rather than RD-TD directions. Figure 1(c) is the corresponding band contrast (BC) image, which shows that deformation twins can be detected in deformed grains. To identify twin types, the special boundary component has been superimposed. In addition, Fig. 1(d) provides the distribution of misorientation angles between neighbouring points in Fig. 1(c). In addition to the low misorientations associated with low angle GBs and noise, there were peaks around 86°, 56° and 38° representing three types of twin boundaries: tension twin (TTW), compression twin (CTW) and double twins (DTW). The dominant twin boundaries were DTW and TTW. Some DTWs and CTWs in this study were not fully indexed by EBSD due to high lattice distortion, and DTWs were determined by the DTW boundary fragments along deformation twin bands. For example, the DTW boundary fragments highlighted in the subsequent high magnification images. Additionally, the thick deformation bands with dark band contrast in Figs 1(c) and S1(a) (Fig. S1(a) in Supplementary File) consisting of fragments of DTWs and CTWs can be treated as shear bands as reported in the literature^4,5^. For example, Basu *et al*. described a twinning-related shear banding, which occurred in areas subjected with intensive CTWs and DTWs^21^. Sandlobes *et al*. also reported that shear bands consisted of large amounts of narrow bands containing the CTWs and DTWs orientation using EBSD and high-resolution orientation TEM^5^.

### Grain nucleation and growth

**Figure 2:**
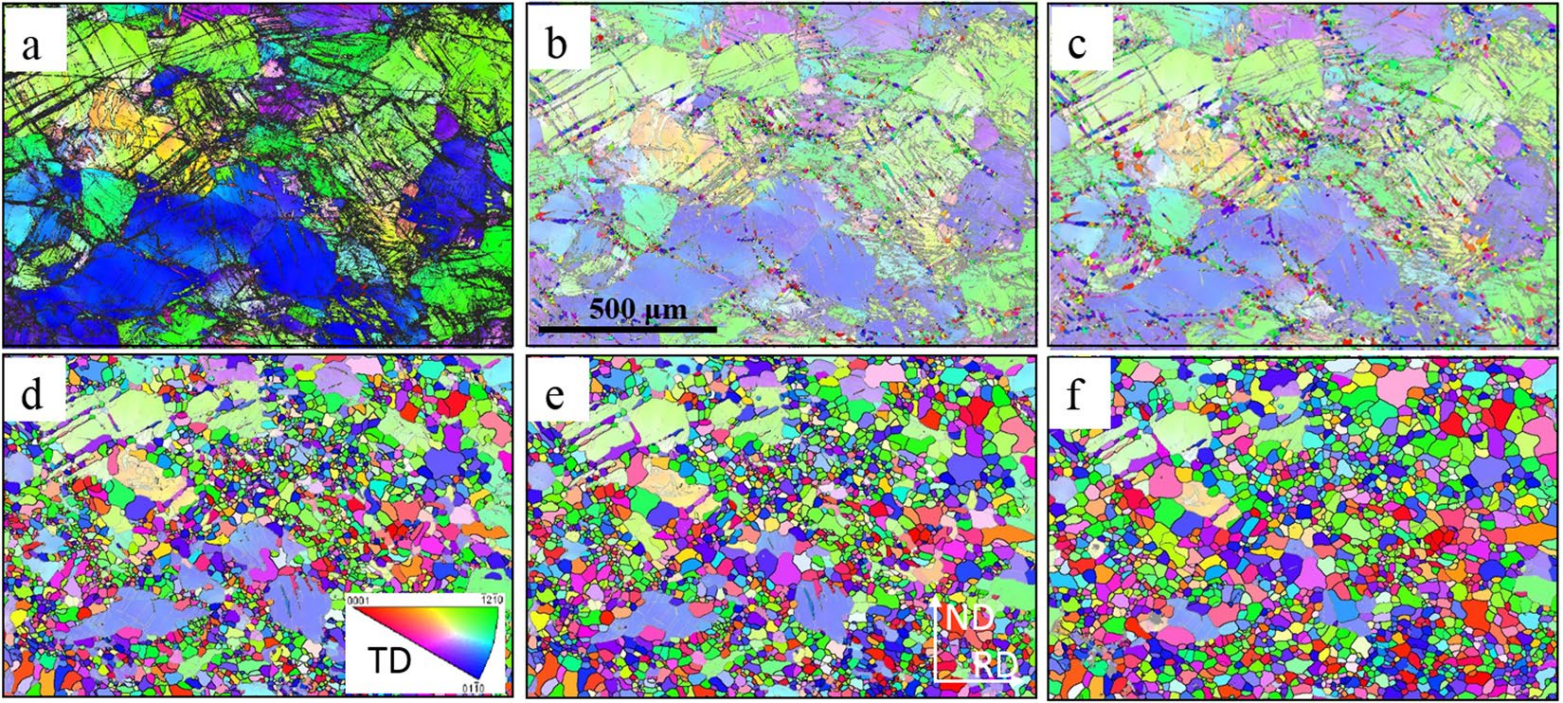
(**a**) EBSD IPF maps after annealing the cold-rolled alloy, only showing recrystallised at annealing intervals of (**a**) 180 s, (b)520 s, (**c**) 880 s, (**d**) 2110 s, (**e**) 3310 s, and (**f**) 6910 s. Observation along TD was applied to IPF triangle.

**Figure 3:**
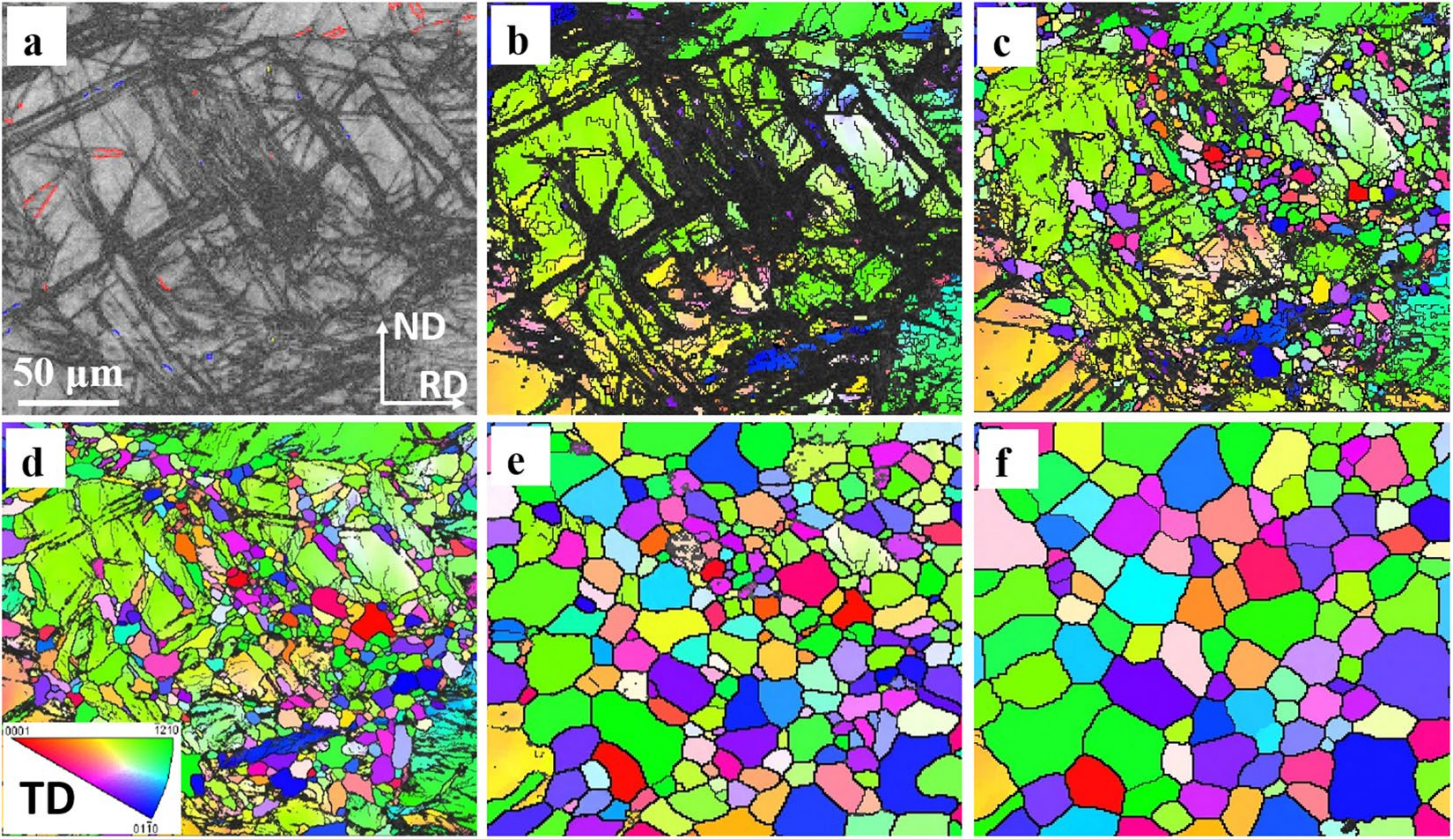
*Quasi-in-situ* EBSD IPF maps presenting recrystallised grain nucleation and growth. (**a**) cold-rolled sample:band contrast (BC) map superimposed by various twin boundaries (see twin boundary type colour codes in (Fig. 1), (**b**) corresponding cold-rolled sample EBSD IPF image and at annealing intervals of (**c**) 520 s, (**d**) 880 s, (**e**) 2110 s, and (**f**) 6910 s. Observation along TD was applied to IPF triangle.

Figure 2(a) shows that no noticeable recrystallisation occurred at the same sampling area in Fig. 1 after annealing for 180 s at 350 °C. Similarly, Fig. 2(b–f) tracks the entire recrystallisation process from nucleation (after annealing 520 s) to nearly full recrystallisation (after annealing 6910 s). This set of *quasi-in-situ* EBSD images display the origin of the recrystallised grains and their subsequent growth. GBs and subgrain boundary migration, second phase particles, deformation twins and shear bands are commonly recognised as nucleation sites for recrystallisation in Mg alloys^4,12,21,36,37^. Recrystallisation initiated by second phase particles does not need to be considered because of the absence of these features before cold rolling and annealing in this study. Although recrystallisation through GB and subgrain boundary migration cannot be completely excluded, careful tracking indicated that the main and preferential nucleation sites were DTWs, DTW-GB (grain boundary) intersections, and shear bands but not TTWs, which agreed well with the previous results of WE43 alloy^11,35^. To further support this, typical high magnification *quasi-in-situ* EBSD images from regions marked by red and yellow rectangles shown in Fig. 1(a) are listed in Figs 3 and S1. The centre deformed grain in Fig. 3 contained a high density of shear bands and DTWs fragments. After annealing at 520 s, a large number of small recrystallised grains appeared at these intensively strained regions. After annealing at 880 s, recrystallised grains started to grow out of shear bands and twin boundaries and consume the adjacent deformed parent grain. During subsequent annealing after 2110 s and 6910 s, grain growth consumed residual deformed regions as well as some small recrystallised grains dominated by the subsequent recrystallisation process. Figure S1 shows similar recrystallisation behaviour to that in Fig. 3. Another purpose of Fig. S1 was to confirm that TTWs (twin boundaries marked in red in Fig. S1) did not recrystallise during the entire recrystallisation process and were gradually consumed by other recrystallised grains during their growth.

### Recrystallised texture evolution

**Figure 4:**
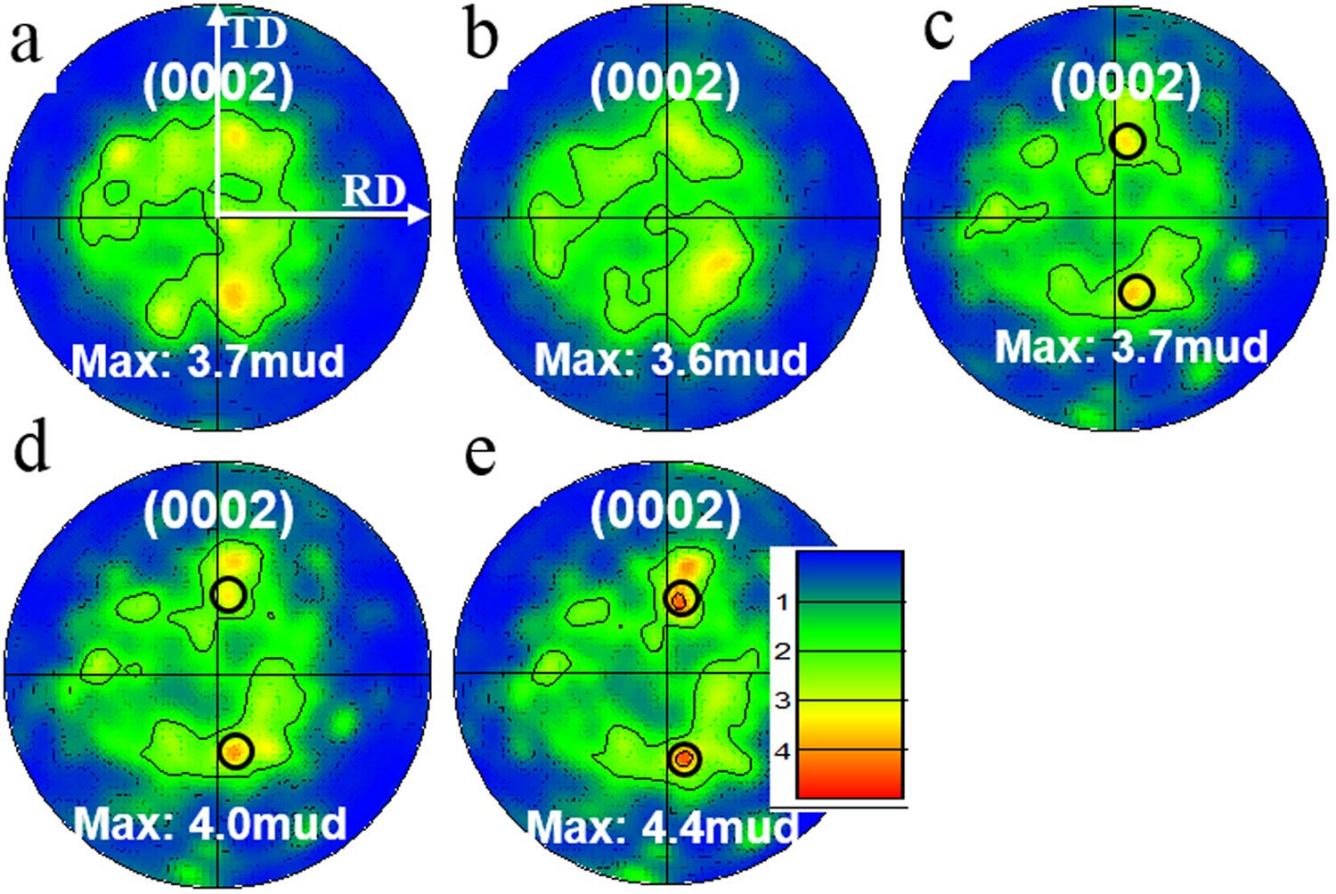
(0002) pole figures consisting only of recrystallised grains at annealing intervals of (**a**) 520 s, (**b**) 880 s, (**c**) 2110 s, (**d**) 3310 s, and (**e**) 6910 s. The axes systems are all the same as shown in (**a**) and legend is shown in (**e**).

Figure 4(a–e) are (0002) pole figures using data collected only from recrystallised grains in Fig. 2. Figure 4(a,b) show pole figures after annealing at 520 s and 880 s where significant nucleation of recrystallised grains occurred. Weakened textures with a peak intensity of 3.7 and 3.6 mud were produced compared to the deformed texture (Fig. 1(e)) of 19.8 mud. Moreover, the texture component distribution in Fig. 4(a,b) was different from the deformed texture (Fig. 1(e)). Figure 4(a) shows a basal texture in the middle of the (0002) pole figure. However, the basal texture faded after annealing for 880 s and two texture components tilted toward the TD direction were produced, Fig. 4(b). With further annealing to 2110 s, 3310 s and 6910 s, the intensity of the texture components around the basal texture gradually decreased and the blue area indicating low texture intensity continued expanding (Fig. 4(c–e)). In contrast, the two peak texture components around 30–34° towards the TD directions, as shown in Fig. 4(b), strengthened throughout the annealing process. The peak texture intensity was increased from 3.6 mud to 4.4 mud. Moreover, a commonly observed RE texture was formed when the alloy was annealed for 2110 s and enhanced with subsequent annealing, Fig. 4(a–e). To find out whether this RE texture was reproducible in this alloy, another sample from a different part of the cold-rolled slab was fully recrystallised and scanned by EBSD. Figure S2 shows this sample consisted of fully recrystallised grains in a large scanning area and its corresponding figure indicates this sample had a non-basal texture and contained RE texture components, which was similar to the recrystallised texture of Fig. 4 and confirmed that this type of recrystallised texture was reproducible in different places of this alloy.

To find out the reason why the RE texture component was enhanced during the grain growth stage in this sample, the corresponding EBSD data within these texture components in the same area in (0002) pole figures were extracted: the peak texture components collected from the two black circles marked in Fig. 4(c–e)). Figure S3 shows the corresponding *quasi-in-situ* EBSD data. Overall, the volume fraction of these texture components in the area that had fully recrystallised (V_r_), excluding the residual deformed area, decreased with annealing (Fig. S3(d–f)). The texture shape and position did not change significantly. The only difference was the texture intensity of the peak texture increased slightly.

### Evolution of individual texture components during annealing

Figure S4 plots the distribution of <0001> directions corresponding to each grain orientation. After cold rolling (Fig. S4(a)) there were three peaks located at 7.5°, 22.5° and 32.5° away from ND. After annealing, the texture component in the range of 70–90° was significantly increased and the peak texture component shifted to 30–47.5° tilted away from ND compared with the cold-rolled sample shown in Fig. S4(a). Moreover, the peak relative frequency decreased from ~0.045 after cold rolling to ~0.025–0.030 after different annealing intervals.

To identify whether some texture components readily grew during static recrystallisation, the recrystallised grains were grouped into four parts, as described in previous studies^18^. These four parts were 0–20° (TCA), 20–45°(TCB), 45–70°(TCC) and 70–90°(TCD) tilted away from ND.

**Figure 5:**
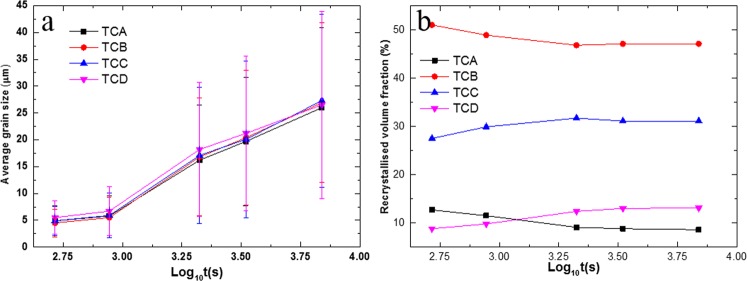
(**a**) Average grain size of each recrystallised texture component and (**b**) recrystallised volume fraction of each texture component based only on the recrystallised area as a function of annealing time.

Figure 5(a) shows the average grain size of recrystallised grains of each texture component. In general, there was no evident difference in grain size for any orientation, i.e. TCA, TCB, TCC and TCD had nearly the same grain size.

Figure 5(b) gives the volume fraction of the individual recrystallised texture components based on total recrystallised area. The volume fractions of TCA and TCB slightly decreased while TCC and TCD increased, especially during the early stage of annealing. Combining the results of Fig. 5(a,b), a conclusion can be made that there was no significant orientated grain growth occurring during annealing, though RE texture was formed in this alloy.

### Concurrent precipitation during recrystallisation

**Figure 6:**
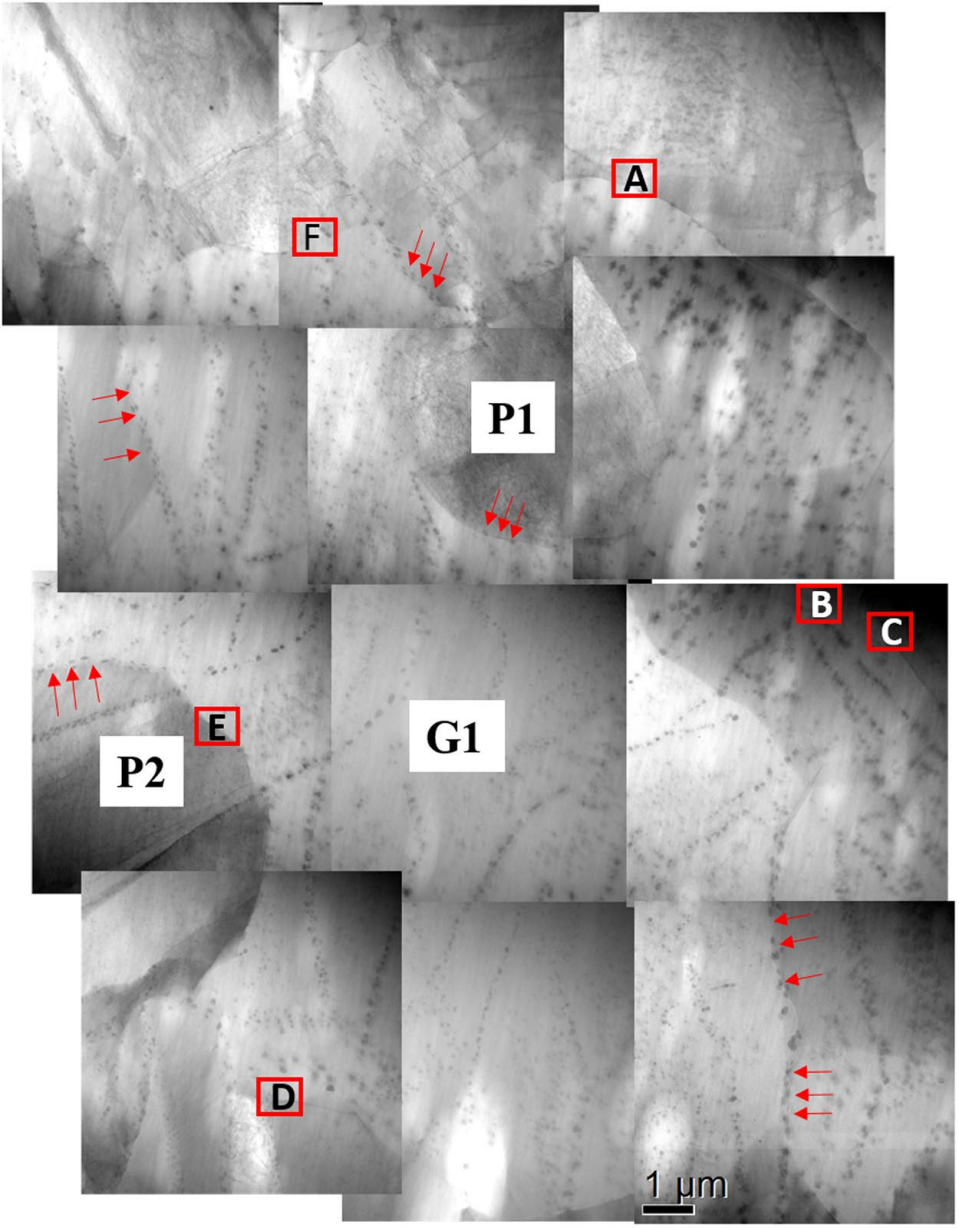
Stitched STEM BF image of a region of interest from cold-rolled alloy after annealing at 350 °C for 880 s.

Figure 6 shows a stitched STEM bright field (BF) image containing 12 single micrographs of alloy ZX10 after annealing at 350 °C for 880 s. Small precipitates can be easily observed in this area. Most precipitates were distributed along pre-existing twin boundaries in the un-recrystallised areas. For example in Fig. S6(a), the residual compression twin (yellow line) and double twin (blue line) fragments in grain P2 were decorated with second phase particles introduced by concurrent precipitation, which can be correlated with grain P2 in Fig. 6. This can be further supported by our previous findings in a WE43 alloy, where precipitates were preferably distributed along twin boundaries after annealing^38^. The precipitates can be also easily observed in recrystallised grains such as in grain G1, Fig. 6. The reason for this is that twin boundaries existed in the prior deformed parent grain, which disappeared when the twins were consumed by the recrystallisation, but second phase particles introduced by concurrent precipitation remained. This phenomenon was reported in the WE43 alloy in our previous studies. The precipitates preferably distributed along prior twin and grain boundaries largely existed during recrystallisation and re-dissolved into the matrix when the samples were almost fully recrystallised^18,35,38^. Figure S5 shows precipitates also distributed along dislocations. Moreover, grain G1 was in the process of growth, but most of its GBs were curved and concave. These concave boundaries were strongly pinned by a string of precipitates parallel to the corresponding grain boundary segment (marked by red arrows). Therefore, the movement of these boundaries was inhibited while the other unaffected or less affected boundaries kept moving forward during grain growth, which resulted in the unusual shape of the recrystallised grain G1.

### Solute segregation along various GBs

**Figure 7:**
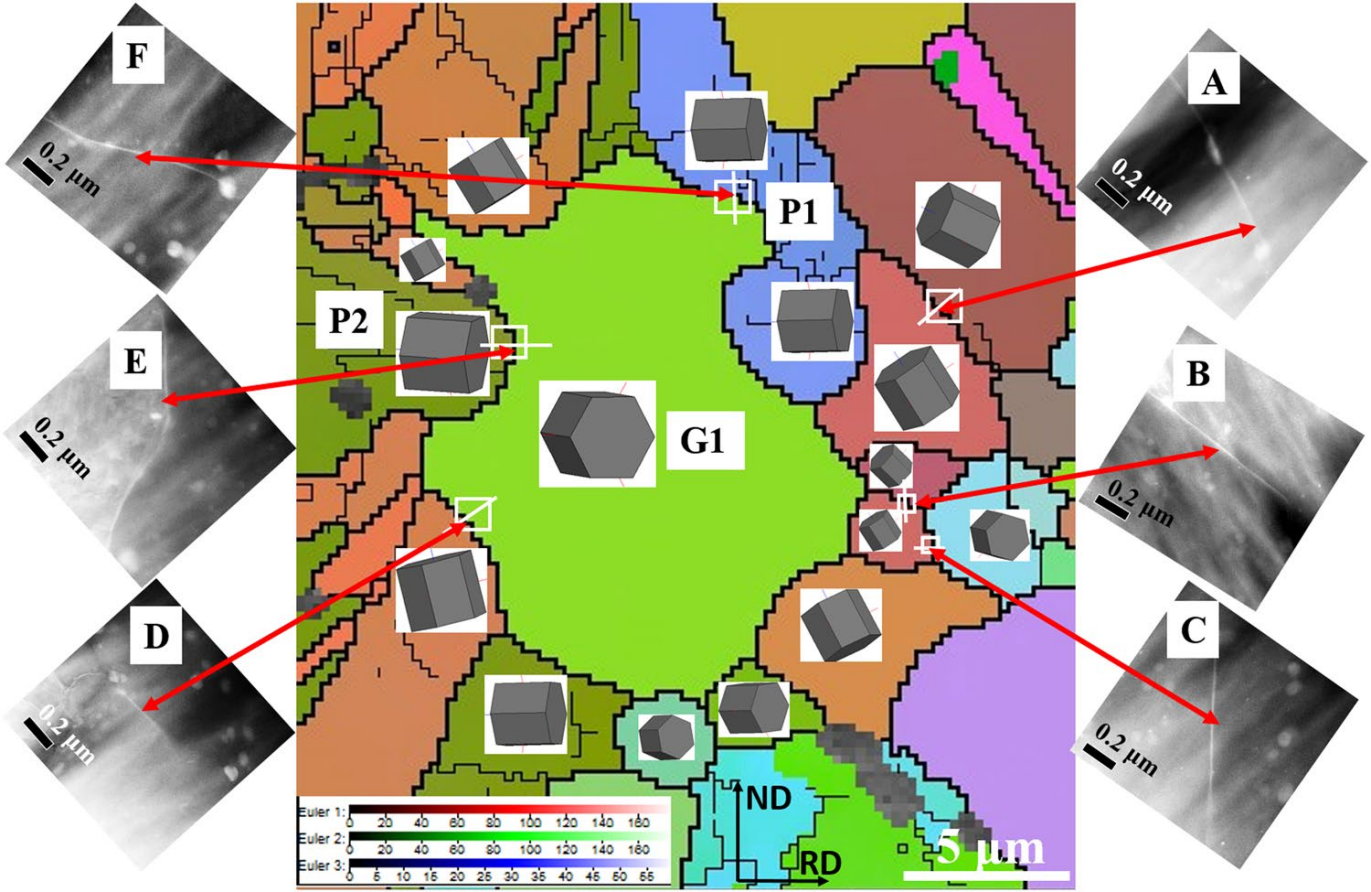
EBSD all Euler map of the investigayted TEM region. The 6 insets (**A**–**F**) HAADF-STEM images were collected from the areas A-F marked in Fig. 6.

In this study, co-segregation of Zn and Ca along GBs was observed using the combination of HAADF-STEM and EDX techniques. In the same region of interest of Fig. 6, 6 GBs marked by red squares and letters A-F were magnified and further investigated by the HAADF-STEM technique. The corresponding high magnification HAADF-STEM images are shown in Fig. 7. All 6 GBs in Fig. 7 exhibited bright contrast in the HAADF mode, meaning that there was segregation on all GBs, with segregation dominated by alloying elements with higher atomic number, i.e., Zn or Ca or both.

To explore the grain boundary types, the crystal orientations of two grains sharing the same grain boundary needs to be determined. An ideal sample area and extensive work are required to determine the misorientation parameters of various GBs if using TEM. Robson *et al*.^22^ and Hadorn *et al*.^39^ observed Y and Ni segregated along a number of different GBs using HAADF-STEM with high-efficiency EDX, but did not provide the exact misorientations of GBs. In this work, the investigated TEM sample as presented in Fig. 6 was transferred into the SEM-EBSD system after finishing TEM investigations. The same region of the TEM work was located and scanned by EBSD, as shown in Fig. S6. The TEM and EBSD maps were correlated by locating the same deformed P1 and P2 grains, and the recrystallised grain G1 (Figs 6, S6 and 7). It should be noted the TEM map needs to be rotated clockwise ~35° to match the EBSD map. Figure S6(a) gives a band contrast EBSD map superimposed with twin boundary types and some twin fragments that remained owing to partial recrystallisation. Figure S6(b) shows a KAM map, which clearly identifies the areas that are un-recrystallised or recrystallised according to local misorientation value.

Figure 7 shows an EBSD all Euler map of the region of interest superimposed with 3D crystal orientations. All the HAADF-STEM images were located in the corresponding area after correlating Figs 6 and 7. Table 1 lists all 6 GBs misorientation parameters. Except for the low angle grain boundaries (LAGB) defined by 0–15°, all the other high angle grain boundaries (HAGB) were detected and distributed with reasonable gaps between 15° and 90°. However, more detailed investigation with advanced 3D imaging techniques is required to determine whether the level of solute segregation depends on the type of GBs, which is extremely difficult to be measured quantitatively at present. The formation of LAGBs are often reported by the mechanism of polygonization^40^. In Fig. S5 and other TEM images (not presented here), precipitates were found to be favourably formed along dislocations suggesting that solute must segregate to dislocation cores. In addition, Zeng *et al*.,^41^ also reported solute segregated along dislocations in a Mg-0.3Zn-0.1Ca (at.%) alloy after cold rolling and annealing. Therefore, it can be reasonably suggested that the LAGBs formed by dislocations rearrangement was segregated by solutes in this study.

**Table 1:** Grain boundary misorientation parameters measured by EBSD.

Grain boundary	Misorientation angle (°)	Misorientation axis	Offset (°)
A	32.5	$0\bar{2}2\bar{1}$	5.97
B	15.5	$\bar{1}\,\bar{1}20$	5.21
C	86.5	$3\bar{2}10$	1.45
D	78.5	$10\bar{8}\,\bar{2}3$	1.53
E	49.8	$10\bar{1}0$	5.20
F	52.4	$\bar{4}\,\bar{1}50$	1.52

**Figure 8:**
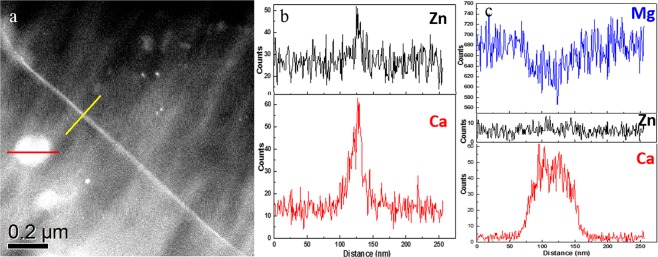
(**a**) A typical HAADF-STEM image from cold-rolled alloy after annealing at 350 °C for 880 s, EDX line scans showing (**b**) solute segregation to a grain boundary and (**c**) element enrichment in a big precipitates.

Figure 8(a) shows a HAADF-STEM image from the same TEM sample. Figure 8(b,c) present the EDX line scan across a grain boundary and a particle. The results indicated Zn and Ca co-segregated along GBs. The particles mainly consisted of Mg and Ca, though a small peak of Zn can be observed within the particle.

## Discussion

In this study, TTWs, DTWs that had rapidly transformed from CTWs, and twinning-related shear bands consisting of DTWs and CTWs were the typical deformation microstructures. A high frequency of CTWs and DTWs has also been observed in other Ca containing alloys, such as the binary Mg-Ca alloys ZX11, reported by Lee *et al*.^27,33^ and ZX31 reported by Kim *et al*.^32^, respectively. Moreover, the addition of Y decreased basal stacking fault energy (SFE) of a binary Mg-Y alloy compared with pure Mg^5^. The Mg-Y alloy also contained significant fractions of CTWs and DTWs compared to the pure Mg after deformation. Similarly, Ca has been reported to decrease the basal SFE^42^. The link between lowering basal SFE and triggering CTWs and DTWs has not been clearly set up, but it seems the reduction of basal SFE may be beneficial for the activation of CTWs and DTWs. Further studies are certainly needed to be conducted to reveal the underlying mechanism.

In this work, the preferential nucleation sites were DTWs and twinning-related shear bands consisting of DTWs and CTWs (Figs 2,3 and S1). The prior grain boundary recrystallization was very limited after careful investigation. CTWs and DTWs have been reported to nucleate recrystallised grains with RE orientations^10,43,44^, which was experimentally confirmed in our previous studies^11,18^. In addition, TTWs were not effective nucleation sites due to minimal accumulation of dislocations and consequently low elastic strain energy along mobile twin boundaries^11,18^. Therefore, the RE texture produced during nucleation stages in this alloy can be attributed to DTWs and twinning-related shear bands recrystallisation.

In Fig. 4, the RE texture distributions were maintained during the growth stages. However, the peak texture components had been strengthened while the texture around basal orientations weakened gradually during annealing. Nevertheless, these changes of local texture components did not change the RE texture distributions.

The final recrystallised texture not only relies on the nucleation of grain orientations, but also depends on the subsequent grain growth of nucleated grains^8,9,16,40,45,46^. Clearly, the question is whether the recrystallised grains originating from DTWs and shear bands with RE orientations survive and grow beyond the prior twin boundaries and shear bands and thereby contribute to the final texture. Furthermore, the multiple effects of orientated grain growth, solute drag and particle pinning need to be considered, since these effects have been shown to totally change the final texture from the early recrystallised texture formed during nucleation. For example, Zeng *et al*.^19^ reported weak basal textures in ternary Mg-0.3Zn-0.1Ca, binary Mg-0.4Zn and Mg-0.1Ca (at.%) after short annealing times. Based on their experiments, the authors hypothesised that the solute drag caused by the co-segregation of Zn and Ca was much stronger than the individual effect segregation of Zn or Ca solutes. This weak texture was preserved in Mg-0.3Zn-0.1Ca after full recrystallisation due to strong solute drag effectively restricting the mobility of high energy GBs of recrystallised grains. In contrast, in both binary alloys, the weak texture was gradually replaced by enhanced basal texture during annealing. The authors attributed this to orientated growth of recrystallised grains with the [0001]//ND and $\langle 11\bar{2}\,0\rangle$//RD orientations due to ineffective solute drag. More recently a Mg-RE study by Barret *et al*.^47^ reported the texture components with characteristic RE texture orientations were observed in both pure Mg and Mg-Y during early dynamic recrystallisation (DRX). Grains with these non-basal orientations lost a growth advantage and basal orientated grains dominated during grain growth in pure Mg. However, the initial characteristic RE texture was preserved in the Mg-Y alloy. The simulations in their study showed grain boundary energy and mobility were essential to account for preferred orientation selection during nucleation and growth^48^. The segregation of Y to grain boundaries homogenised the GBs energy and correspondingly decreased the mobility of basal orientated GBs, which largely reduced grain growth of $\langle 10\bar{1}0\rangle \,$ extrusion orientations and allowed other non-basal orientations to be preserved due to equal grain growth^47^.

In Figs 6–8 and Table 1, although only a local area was systematically investigated regarding solute segregation behaviour, direct evidence is provided that solute segregation occurred along a wide range of HAGBs instead of only along special boundary types, at least in this local area. To the best of our knowledge, this study is the first to report coupling HAADF-STEM and EBSD techniques in Mg alloys to clearly explore the relationship between misorientation and the occurrence of solute segregation regarding recrystallisation mechanism. However, due to variations in local concentration of Ca and Zn, the segregation behaviour in Figs 6–8 may not represent the entire sample. A further statistical HAADF-STEM study will certainly warrant more findings of solute segregation behaviour in Mg alloys. Although direct observation of segregation along LAGBs was not provided in this study, solute segregation along LAGBs can be demonstrated indirectly as illustrated in Fig. S5. Thus, it has been shown that solute could segregate along all GBs. After close examination, the volume of recrystallised texture components close to basal orientations including TCB and specifically TCA decreased through the annealing while TCC and TCD increased due to slightly different growth rates. These changes were more significant during the nucleation stage where no significant precipitation occurred and therefore there was no pinning of recrystallised grain growth. Therefore, the solute drag largely restricted the grain boundary mobility of recrystallised basal grains, which would otherwise grow favourably in conventional alloys. Subsequently, after annealing for 880 s the particles formed by concurrent precipitation further facilitated this recrystallisation behaviour. In addition, the precipitates along prior GBs could also reduce the occurrences of recrystallisation along GBs, which were restricted by precipitate pinning. This could further reduce the source of basal recrystallised grains and weaken the basal texture, as illustrated in our previous work^18^.

The texture position and intensity were altered by static recrystallisation, Fig. S4. Overall, the recrystallised texture component distribution was preserved during the whole recrystallisation process. Nevertheless, changes in local distribution need to be noted. For instance, the volume fraction of texture component containing 0–10° tilted away from the ND decreased (marked by black boxes in Fig. S4) and the peak texture components (marked by blue boxes in Fig. S4) slightly increased during annealing, which is consistent with the (0002) pole figure results in Figs 4 and S3. These results further indicated the effect of solute drag and particle pinning on basal grain growth was more prominent than non-basal grains, which made most small basal grains lose growth advantage when they meet relatively large grains with other orientations.

From the results of this study, as well as our previous studies^11,18,35^, and the results from some other Ca or RE containing Mg alloys in the literature^12,19,47^, the typical RE textures were normally formed during the nucleation stage of static recrystallisation or the early DRX stage in dynamic recrystallisation studies. The RE orientated grains survived and dominated the texture evolution. This was because recrystallisation from other sources was retarded due to solute drag and particle pinning from concurrent precipitation, which preserved the RE texture during the entire annealing process. This texture evolution resulting from uniform grain growth of nucleated recrystallised grains is called oriented nucleation instead of orientated growth^32,49^. These results suggested that Ca acted in a similar manner to RE elements during annealing: imparting solute drag, concurrent precipitation, which restricted preferential growth of basal grains.

## Conclusions

The entire recrystallisation sequence of ZX10 was examined using the *quasi-in-situ* EBSD method during annealing, and the following conclusions can be drawn:

1. Solute segregation occurred along a wide range of GB types rather than just special boundaries, at least in the investigated local area in this study. Moreover, it can be reasonably speculated that solute segregation along LAGBs occurred as well.
2. Concurrent precipitation occurred preferentially along prior grain and twin boundaries. These precipitates pinned the prior GBs and reduced the recrystallisation frequency along GBs, which reduced the source of recrystallised grains with a basal texture. In addition, these precipitates pinned some GBs which slowed down the grain growth and restricted potential orientated grain growth, especially the preferential growth of recrystallised grains with basal orientations that occurs in pure Mg or other conventional Mg alloys.
3. DTWs and twinning-related shear bands were the dominant nucleation sites and contributed mainly to the final recrystallised textures. Characteristic RE textures were produced and preserved by uniform grain growth during the whole recrystallisation sequence due to synergic effects of solute drag and particle pinning introduced by concurrent precipitation. These results suggested that Ca played a similar role to that of RE elements in controlling recrystallisation texture. However, changes in local distribution needs to be noted: the RE texture components were enhanced while the texture around basal orientations weakened gradually during annealing. Nevertheless, these local changes did not alter the RE texture morphologies.

## Methods

The as-cast ZX10 was supplied by Magnesium Elektron, with chemical compositions listed in Table 2. The alloy was homogenised at 450 °C for 48 h. Several rectangular plates 50 × 30 × 8 mm^3^ were then cut from the centre of heat-treated sample for subsequent cold rolling.

**Table 2:** Chemical composition of as-received ZX10 alloy.

Alloy	Zn(wt.%)	Ca(wt.%)	Mn(wt.%)	Fe(wt.%)	Mg
ZX10	0.8	0.2	0.02	0.005	Balance

The alloy was cold rolled in one pass to reduction of 20%. The *quasi-in-situ* EBSD sample procedure and scanning experimental details can be found in previous studies^11,18,38^. The EBSD scans of the same area were taken after cold rolling and after 180 s, 520 s, 880 s, 2110 s, 3310 s, and 6910 s annealing at 350 °C with a step size of 1 μm. Another cold-rolled sample from different part of the above mentioned *quasi-in-situ* EBSD sample was fully recrystallised and scanned by EBSD. The recrystallised grains in all EBSD maps were determined by grain orientation spread (GOS) value^16^. Only grains with GOS <1 were identified as recrystallised grains and this criterion agreed well with the experimental results.

A JEOL 2010F microscope operated at 200 kV was used to conduct high-angle annular dark-field scanning transmission electron microscopy (HAADF-STEM) and energy dispersive X-ray (EDX) spectroscopy analysis. To investigate solute segregation along various GBs, a cold-rolled sample was partially recrystallised, annealed at 350 °C for 880 s. A 3 mm disc with the thickness of 100 μm from the middle part of RD-ND plane was produced by traditional grinding. A fine scratch was used to indicate the rolling direction. An electrolyte consisting of 8.8 g lithium chloride, 18.6 g magnesium perchlorate, 840 ml methanol and 160 ml 2-butoxy-ethanol, at −35 °C and 32 V was used for twin-jet thinning. To obtain a contamination-free surface after electropolishing, this disc was finally ion milled using a Gatan PIPSΙΙ at 0.3 kV for 5 minutes. When useful data from a region of interest were collected using HAADF-STEM and EDX techniques, the same sample was transferred into the SEM-EBSD system. This region of interest was located and scanned. To analyse local misorientation within these microstructure, kernel average misorientation (KAM) maps of the EBSD data were produced using a filter size of 3 × 3.

## Supplementary information

Revised supplementary material

## Data Availability

The datasets generated during and/or analysed during the current study are available from the corresponding author on reasonable request.
