# Supplementary material for: Exploring the mechanism of “Rare Earth” texture evolution in a lean Mg–Zn–Ca alloy
Source: Sci Rep. 2019 May 9;9:7152. doi: 10.1038/s41598-019-43415-z (PMC6509330; doi:10.1038/s41598-019-43415-z)
Supplement: Supplementary file 1 — Revised supplementary material [file 41598_2019_43415_MOESM1_ESM.pdf]

Exploring the mechanism of "Rare Earth" texture evolution in a lean Mg–Zn–Ca alloy

Dikai Guan*, Xingguang Liu, Junheng Gao, Le Ma, Bradley P Wynne, Mark Rainforth*

Department of Materials Science and Engineering, University of Sheffield, Sheffield S1 3JD, UK

*Corresponding authors: dikai.guan@sheffield.ac.uk, m.rainforth@sheffield.ac.uk

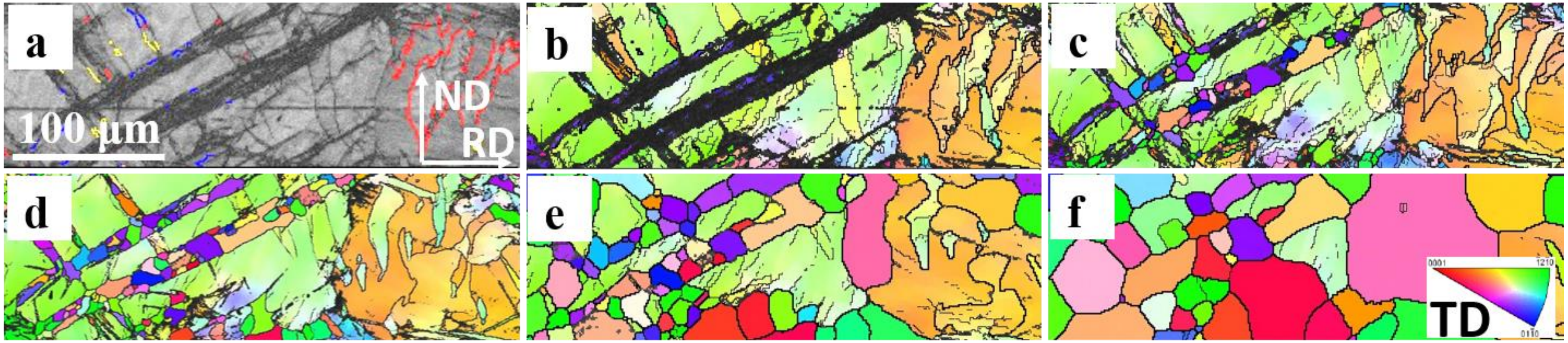

Figure S1 *Quasi-in-situ* EBSD IPF maps presenting recrystallised grain nucleation and growth in ZX10 (a) cold-rolled sample:band contrast (BC) map superimposed by various twin boundaries (see twin boundary type colour codes in Fig. 1), (b) corresponding cold-rolled sample EBSD IPF image and at annealing intervals of (c) 520s, (d) 880s, (e) 2110s, and (f) 6910s. Observation along TD was applied to IPF triangle.

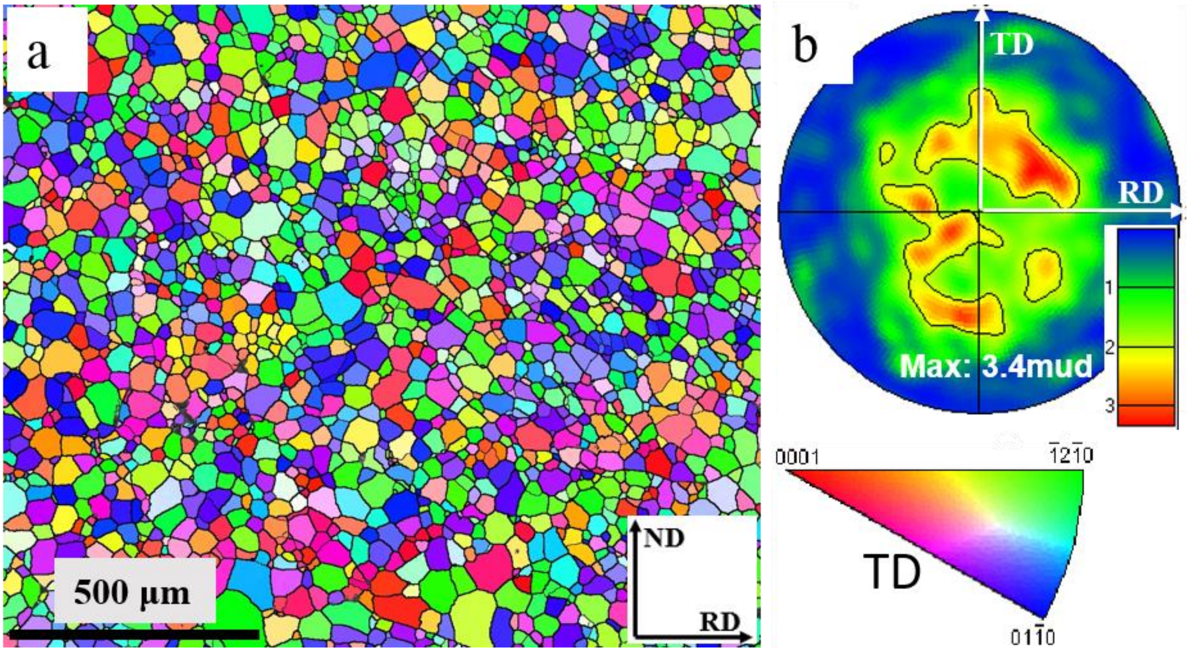

Figure S2 (a) EBSD IPF maps of another fully recrystallised sample from different part of this alloy, and (b) corresponding (0002) pole figure. Observation along TD was applied to IPF triangle.

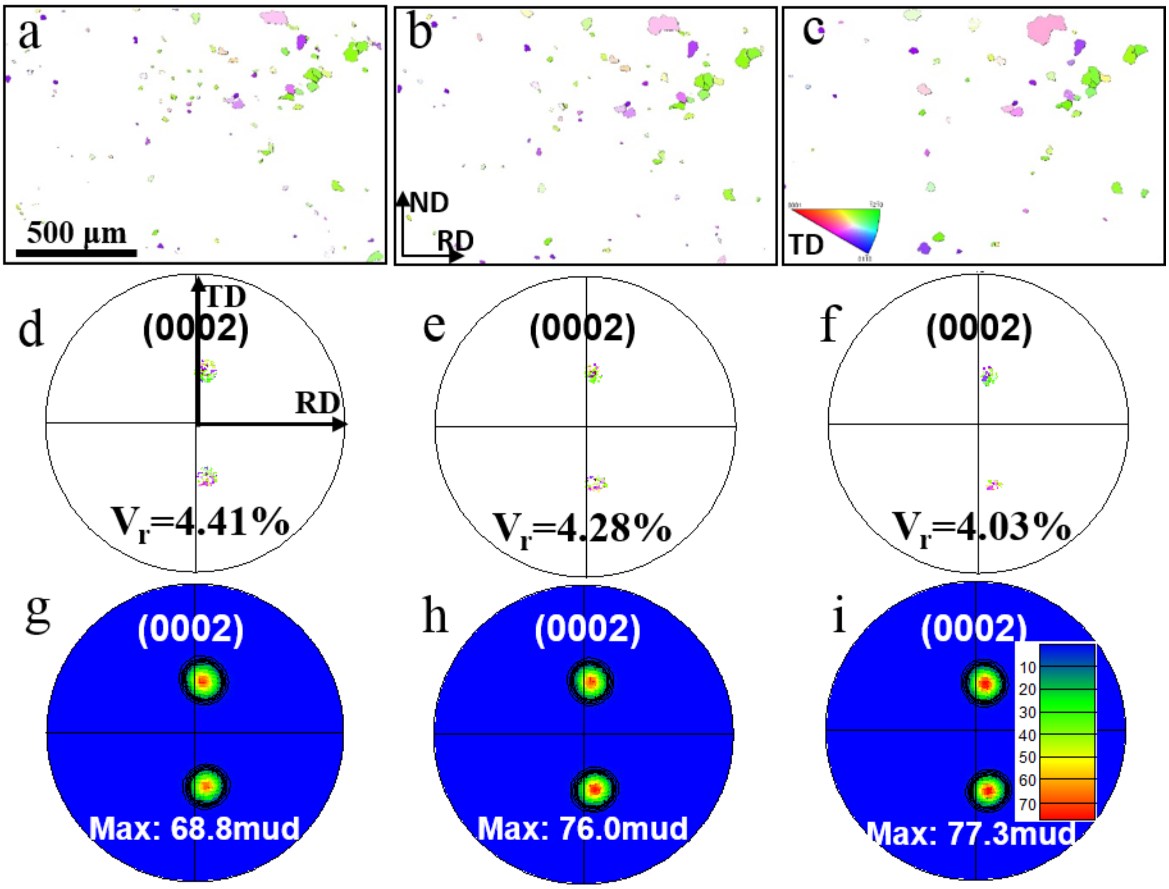

Figure S3 *Quasi-in-situ* EBSD IPF maps presenting recrystallised grains within peak intensity of texture distribution circled in Fig. 5 at annealing intervals of (a) 2110s, (b) 3310s, (c) 6910s. (d-f) are corresponding (0002) scattered point pole figure and (g-i) are corresponding recalculated contour (0002) pole figure. Observation along TD was applied to IPF triangle.

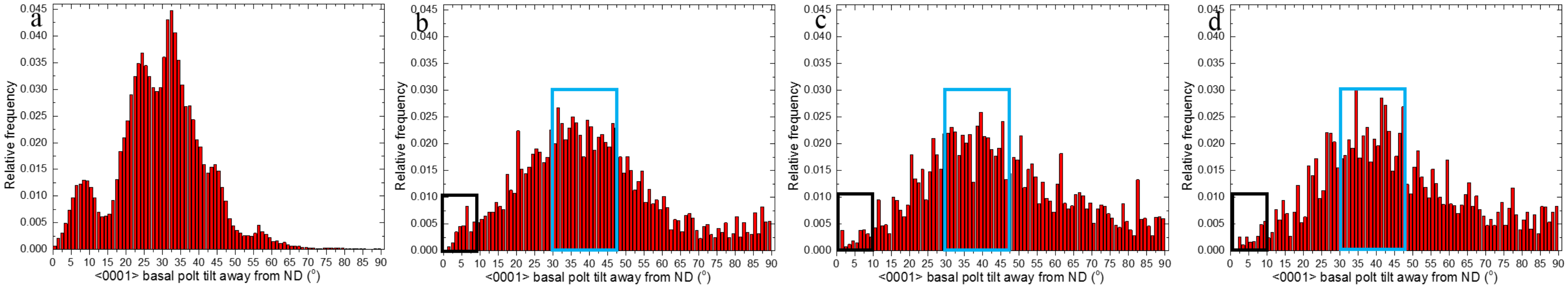

Figure S4 <0001> basal texture tilted away from Normal Direction (ND) at annealing intervals of (a) 0s, (b) 520s, (c) 2110s, (d) 6910s for alloy ZX10 and (e) 0s, (f) 385s, (g) 1030s, (g) 3430s for alloy ZX10.

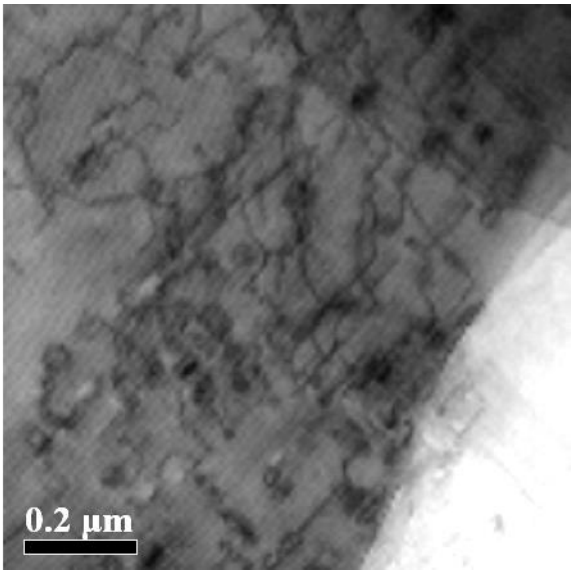

Figure S5 A STEM BF image from cold-rolled ZX10 after annealing at 350 °C for 880s

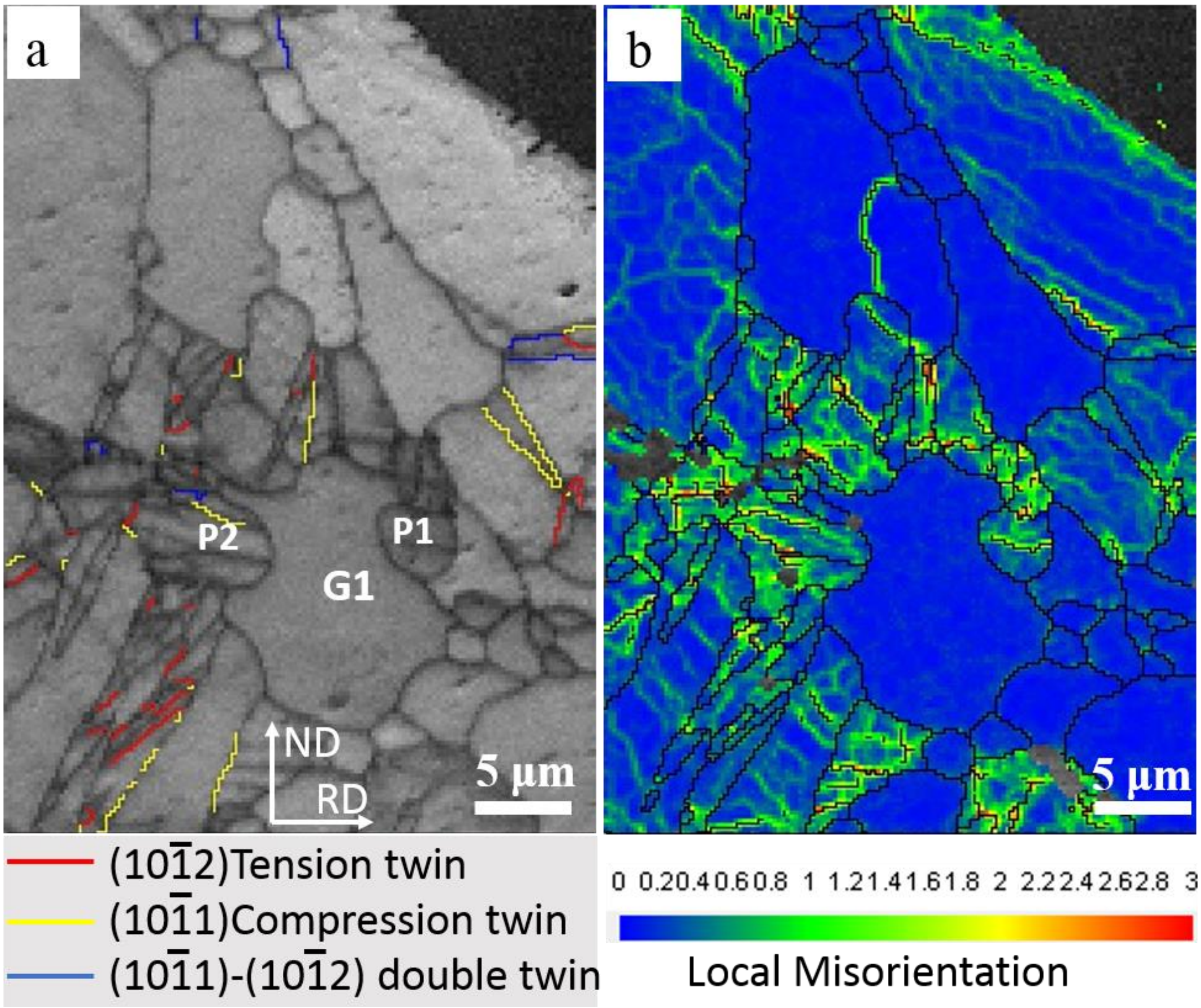

Figure S6 (a) Band contrast (BC) map superimposed by various twin boundaries, (b) corresponding KAM map of the TEM sample shown in Fig. 6.
